# European mtDNA Variants Are Associated With Differential Responses to Cisplatin, an Anticancer Drug: Implications for Drug Resistance and Side Effects

**DOI:** 10.3389/fonc.2019.00640

**Published:** 2019-07-19

**Authors:** Tej H. Patel, Lucas Norman, Steven Chang, Sina Abedi, Catherine Liu, Marilyn Chwa, Shari R. Atilano, Kunal Thaker, Stephanie Lu, S. Michal Jazwinski, Michael V. Miceli, Nitin Udar, Daniela Bota, M. Cristina Kenney

**Affiliations:** ^1^Gavin Herbert Eye Institute, University of California, Irvine, Irvine, CA, United States; ^2^Illinois Eye and Ear Infirmary, University of Illinois at Chicago, Chicago, IL, United States; ^3^VA Medical Center Long Beach Hospital, Long Beach, CA, United States; ^4^Tulane Center for Aging and Department of Medicine, Tulane University, New Orleans, LA, United States; ^5^Department of Neurology, Neuro-Oncology Division, University of California, Irvine, Irvine, CA, United States; ^6^Department of Pathology and Laboratory Medicine, University of California, Irvine, Irvine, CA, United States

**Keywords:** cisplatin, mitochondria, cybrids, drug resistance, mtDNA haplogroups

## Abstract

**Background:** Cisplatin, a powerful antitumor agent, causes formation of DNA adducts, and activation of apoptotic pathways. Presently, cisplatin resistance develops in up to 70% of patients but the underlying molecular mechanism(s) are unclear and there are no markers to determine which patients will become resistant. Mitochondria play a significant role not only in energy metabolism but also retrograde signaling (mitochondria to nucleus) that modulates inflammation, complement, and apoptosis pathways. Maternally inherited mitochondrial (mt) DNA can be classified into haplogroups representing different ethnic populations that have diverse susceptibilities to diseases and medications.

**Methods:** Transmitochondrial cybrids, where all cell lines possess identical nuclear genomes but either the H (Southern European) or J (Northern European) mtDNA haplogroups, were treated with cisplatin and analyzed for differential responses related to viability, oxidative stress, and expression levels of genes associated with cancer, cisplatin-induced nephrotoxicity and resistance, apoptosis and signaling pathways.

**Results:** The cisplatin-treated-J cybrids showed greater loss of cell viability along with lower levels of reactive oxygen species and mitochondrial membrane potential compared to cisplatin-treated-H cybrids. After cisplatin treatment, J cybrids showed increased gene expression of *BAX, CASP3*, and *CYP51A*, but lower levels of *SFRP1* compared to untreated-J cybrids. The cisplatin-treated-H cybrids had elevated expression of *CDKN1A/P21*, which has a role in cisplatin toxicity, compared to untreated-H cybrids. The cisplatin-treated H had higher transcription levels of *ABCC1, DHRS2/HEP27*, and *EFEMP1* compared to cisplatin-treated-J cybrids.

**Conclusions:** Cybrid cell lines that contain identical nuclei but either H mtDNA mitochondria or J mtDNA mitochondria respond differently to cisplatin treatments suggesting involvement of the retrograde signaling (from mitochondria to nucleus) in the drug-induced cell death. Varying toxicities and transcription levels of the H vs. J cybrids after cisplatin treatment support the hypothesis that mtDNA variants play a role in the expression of genes affecting resistance and side effects of cisplatin.

## Introduction

Cisplatin is a non-specific alkylating agent used for decades to successfully treat various cancers. However, cisplatin has dose-dependent toxicity and resistance often develops (1, 2). Mitochondrial dynamics play an important role in resistance to chemotherapy and severity of side effects. Many anti-cancer medications can cause mitochondrial dysfunction and DNA damage, and cells depleted of mtDNA show increased resistance to chemotherapeutic agents. Cisplatin is a pro-apoptotic drug that damages mitochondria, contributing to the toxicities in gastrointestinal, auditory, and kidney proximal tubule cells (3–5). Cisplatin treatment of head and neck squamous cell carcinoma and Chinese hamster ovarian cells significantly increases adduct formation in mitochondrial (mt) DNA compared to nuclear (n) DNA (6). Furthermore, mtDNA is more likely to remain damaged after cisplatin exposure because mitochondria lack the nucleotide-excision repair mechanisms found in nDNA. The severity of cancer drug side effects and incidence of induced resistance to chemotherapy drugs vary amongst individuals but the mechanism(s) are still not fully understood.

Cisplatin side effects include nausea, vomiting, myelosuppression, nephrotoxicity, neurotoxicity, cognitive dysfunction retinopathy, and hearing loss. Intravenous delivery of cisplatin commonly causes mild to moderate pigmentary retinopathy, along with intra-retinal hemorrhages, exudates, and cotton wool spots (7). In addition, significant vision loss can occur with both systemic and local delivery methods of the drug (7). However, it is unclear which individuals are going to be susceptible to this toxicity. The decision to continue a drug regimen depends not only on the effectiveness in treating the malignancy, but also on a person's tolerance to the drug and the risks of end-organ damage. Thus, insight into the mechanisms of cisplatin toxicity is valuable to patient care and therefore, a model using human retinal pigment epithelial (RPE) cells was developed to study mechanisms of cytotoxic damage.

Mitochondria are unique organelles that play essential roles in ATP production, calcium homeostasis, apoptosis, and cell signaling. The mtDNA are maternally inherited and can be classified into different haplogroups based on patterns of single nucleotide polymorphisms (SNPs) that have accumulated over thousands of years. The mtDNA haplogroups represent populations from different geographic origins and subsequently, can be used to characterize different ethnic groups. Clinically, different ethnic populations show dissimilar susceptibilities to diseases and drug responses (8–10), and it has been suggested that mtDNA haplogroups may play important roles in these differences (11). Specific mtDNA haplogroups have also been associated with renal, prostate, breast, and lung cancers (12–15). In addition, somatic and germline mtDNA mutations, as well as levels of mtDNA copy numbers, have been associated with increased risk of cancer and different responses to anti-cancer drugs (16). These studies demonstrate how polymorphisms and/or variants in mtDNA can lead to significant changes at the molecular and cellular levels and can be associated with increased cancer risk.

One method to characterize the functional consequences of cells having specific mtDNA haplogroups is through transmitochondrial cybrids (cells with identical nuclei but different mtDNA). Previously, we demonstrated that H cybrids (Southern European maternal origin mtDNA) vs. J cybrids (Northern European maternal origin mtDNA) have significantly different cellular homeostasis. Although all cybrids had identical nuclei and culture conditions, cells containing the J mtDNA had increased rates of growth along with higher lactate and glycolysis levels, but showed significantly lower MT-RNA expression and ATP levels compared to the H cybrids (17, 18). Interestingly, even though the J cybrids were created in a non-cancerous human retinal cell line (ARPE-19), the behavior of these cells was characteristic of the *Warburg Effect*, which described cancerous cells to be more glycolytic, using less oxidative phosphorylation, and producing high amounts of lactate in the presence of oxygen (aerobic glycolysis).

Using a HeLa cybrid model, Amo et al. was able to demonstrate that resistance to cisplatin was conferred via alterations of the mtDNA within the control region and that cisplatin-resistant clones possessed shorter OriB variants within a 16184-16193 region enriched with cytosine repeats (19). Interestingly, alterations of the nuclear genome were not involved in the cisplatin resistance. These findings support the hypothesis that the SNP differences that define the different haplogroups would be important for the responses to cisplatin and as the H and J haplogroup populations possess disparate SNP patterns, then their cybrids would show different responses.

In the present study, we compared the effects of cisplatin on human RPE cell cybrids that possessed either H or J haplogroup mtDNA and found differential responses in levels of cell viability, reactive oxygen species production, mitochondrial membrane potential, and gene expression levels in pathways related to cell signaling, apoptosis, and cisplatin resistance.

## Methods

### Cybrid Creation

**Figure 1:**
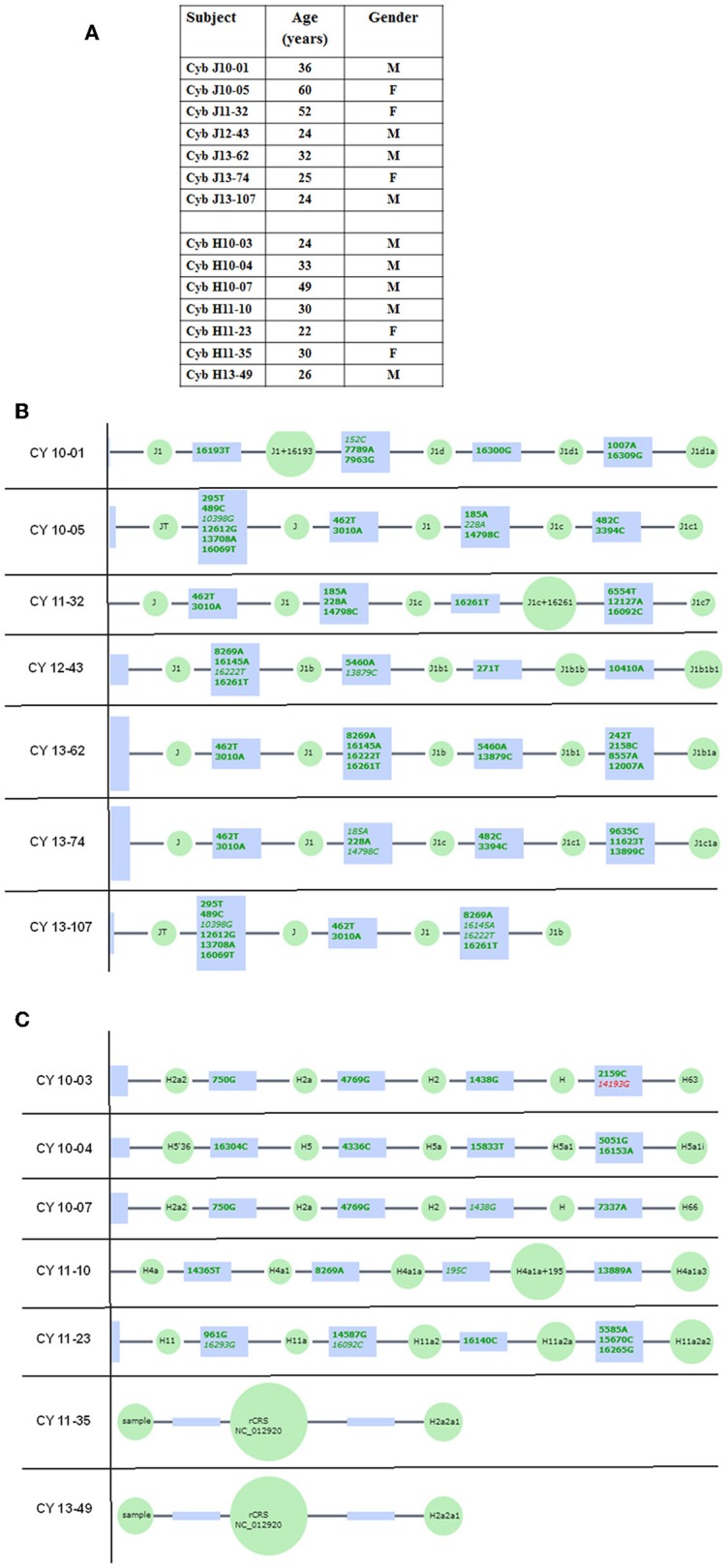
**(A)** Age and gender profiles for the J haplogroup (*n* = 7) and H haplogroup (*n* = 7) subjects. **(B)** Schematic derived from Haplogrep (https://haplogrep.uibk.ac.at) showing the SNPs that define the J mtDNA haplogroups for subjects used in this study. **(C)** Schematic derived from Haplogrep (https://haplogrep.uibk.ac.at) showing the SNPs that define the H mtDNA haplogroups for subjects used in the study. Red font, Haplogroup associated polymorphism missing. Green font, Polymorphisms associated with haplogroup.

All subjects read and signed an informed consent (IRB #2003-3131) from the Institutional Review Board of the University of California, Irvine. All clinical investigations and protocols were conducted according to the principles of the Declaration of Helsinki and approved by the appropriate investigational review boards (University of California, Irvine). Cybrids were generated as described previously (17, 18). H and J cybrids were created by polyethylene glycol fusion of platelets with the Rho*0* (mtDNA free) ARPE-19 cells, which had been treated by low dosage ethidium bromide as described by Miceli and Jazwinski (20). H and J cybrids were cultured to the fifth passage using DMEM-F12 containing 10% dialyzed fetal bovine serum, 100 unit/ml penicillin and 100 μg/ml streptomycin, 2.5 μg/ml fungizone, 50 μg/ml gentamycin, and 17.5 mM glucose. Figure 1 provides description of the background of the subjects used in this study. The ages for the H mtDNA subjects (*n* = 7 cybrids) were 30.57 ± 3.39 years old, while ages for the J mtDNA subjects (*n* = 7 cybrids) were 36.14 ± 5.47 years (*P* = 0.4) (Figure 1A). There were 4 males and 3 females for the J haplogroup subjects and 5 males and 2 females in the H haplogroup subjects. Figures 1B,C show the haplogroup defining SNPs in the mtDNA for the J cybrids and H cybrids used in this study (see sequencing method below).

### Sequencing of mtDNA From H and J Cybrids

DNA was extracted from the individual cybrids (*n* = 7 for H cybrids and *n* = 7 for J cybrids) using a kit (DNeasy Blood and Tissue Kit, Qiagen, Germantown, MD). Next Generation Sequencing (NGS) technology that sequences both strands of mtDNA independently in both directions was used to quantitate the haplogroup-defining SNPs, private SNPs (not defining haplogroups), and low frequency heteroplasmy SNPs across the entire mitochondrial genome.

#### NGS Sequencing

Primers were designed across the entire human mitochondrial genome. A total of 171 primer pairs were designed that overlapped each other to capture the intervening sequences. A total of 100 ng of DNA per sample was used to construct NGS libraries using the TruSeq Custom Amplicon kit (Illumina, San Diego, CA). Two independent sets of primer pools were synthesized. The two primer pools had primers that were complementary. The two primer pools would interrogate the two strands of the mitochondrial genome independently. The two independent libraries had 48 samples each. NGS sequencing was done using 96 libraries (for 48 samples X 2 pools) that were loaded in 2 lanes for a 2 × 225 run on a HiSeq instrument (Illumina). After sequencing, somatic variant analysis was carried out using the variant caller—Pisces (21). Variant calls from each pool were combined to get a list of “true” variants. True variants are defined as variants present on both strands of the DNA. The true variant list was used for final haplogroup analyses using Haplogrep (http://haplogrep.uibk.ac.at). Variants present on only one strand may represent DNA modification events or other artifacts especially at low frequency and therefore were eliminated from the final haplogroup analysis. This method is capable of deep sequencing (average sequencing depth of 30,000; range 1,000–100,000) and accurately differentiates low frequency mtDNA heteroplasmy SNPs from DNA modification artifacts. The amino acid changes and association with diseases resulting from some of the SNPs variants were verified using www.MitoMap.org (22, 23). In some cases, the www.Phylotree.org (24) was used to verify the positions of specific SNPs within the Haplogroup H or Haplogroup J, and also determine if the SNPs were elsewhere in the entire mtDNA tree Build 17. The rs numbers were identified using www.ncbi.nlm.nih.gov/snp. The website www.hmtvar.uniba.it, a database with over 40K human mtDNA variants (25), was utilized to search for specific variants using the Query Tab and inputting the SNP value into the Position field to determine Mutation, Amino Acid Change (if any), and Locus, as well as the link to external resources such as dbSNP for rs numbers. All of the SNPs identified had a Quality Score of 100 (A Phred-scaled quality score assigned by the variant caller) and PASSed all of the Filters.

### IC-50 Analysis of Cisplatin Titration Curve Measuring Cell Viability

H and J cybrids were plated in 96-well plates (10,000 cells/well), incubated for 24 h, and then treated with 0, 20, 40, 60, 80, 100, or 120 μM of cisplatin. The cybrids were incubated for another 48 h before having their cell viabilities measured with MTT reagent (Cat. # 30006, Biotium, CA) and absorbance measured with an ELx808 spectrophotometer (Biotek) at 570 nm with reference wavelength at 630 nm. The background absorbance was subtracted from the signal absorbance and values normalized to the untreated specimen of each cell line. Each treatment was analyzed with eight replicates. An IC-50 analysis was performed to determine the concentration of cisplatin required to inhibit the cell viability by 50% (GraphPad Prism Software, Inc., San Diego, CA).

### Cell Viability Assay

H and J cybrids were plated in six-well plates (500,000 cells/well) and incubated for 24 h. Then cisplatin was added to media at concentrations of 0, 25, or 50 μM and incubated for another 48 h. Cells were counted using a Cell Viability Analyzer (ViCell, Beckman Coulter, Miami, FL) that incorporates a Trypan blue dye exclusion method. Cell numbers were normalized to H untreated and analyzed using the two-tailed *t*-test (GraphPad Prism). Experiments were analyzed in triplicate replicates and the entire experiment was repeated twice.

### Mitochondrial Membrane Potential (ΔΨm) Assay

H and J cybrids were plated in 24-well plates (100,000 cells/well) were incubated for 24 h and treated with 0 or 40 μM of cisplatin for another 48 h. JC-1 reagent (5,5′,6,6′-tetrachloro-1,1′,3,3′- tetraethylbenzimidazolylcarbocyanine iodide) (Biotium, Hayward, CA) was added to cultures for 15 min. Fluorescence was measured using a Gemini XPS Microplate Reader (Molecular Devices) for red (excitation 550 nm and emission 600 nm) and green (excitation 485 nm and emission 545 mm) wavelengths. Intact mitochondria with normal ΔΨm appeared red, while cells with decreased ΔΨm were in a green fluorescent state. Experiments were analyzed in quadruplicate and the entire experiment was repeated three separate times. Cisplatin-treated values were compared to untreated values for statistical significance (*P* ≤ 0.05, GraphPad Prism Software, Inc.).

### Reactive Oxygen/Nitrogen Species (ROS) Assay

H and J cybrids were plated in 24-well plates (100,000 cells/well) and incubated for 24 h. Cells were treated with 0 or 40 μM of cisplatin for another 24 or 48 h. ROS levels were measured with fluorescent dye 2,7-dichlorodihydrofluorescin diacetate (H_2_DCFDA, Invitrogen-Molecular Probes, Carlsbad, CA) on a fluorescence plate reader using 490 nm for emission and 520 nm for excitation wavelengths (Gemini XPS Microplate Reader, Molecular Devices, Sunnyvale, CA).

Our previous studies have shown that the J cybrids grow more rapidly than H cybrids (17) so the ROS levels were normalized per cell number. Simultaneously to the ROS experiments, H and J cybrids were plated in 6 well-plates (500,000 cells/plate), incubated 24 or 48 h, and treated in the identical fashion as described above. Cell viabilities were assessed by the Beckman Coulter ViCell Counter, allowing us to determine ROS levels per cell numbers. Differences in cisplatin-treated cells compared to untreated cells were analyzed (Prism, GraphPad Software Inc.) and were considered to be statistically significant when *P* ≤ 0.05. Experiments were analyzed in quadruplicate replicates and the entire experiment repeated three separate times.

### RNA Isolation, cDNA Synthesis, and Quantitative Reverse Transcription PCR (qRT-PCR)

H and J cells were plated (500,000 cells/well) and incubated for 24 h in six-well plates. Cells were treated with culture media containing either 0 or 40 μM of cisplatin for another 48 h. Trypsinized cells were pelleted and RNA isolated following the manufacturer's protocol (RNeasy Kit, Qiagen, Valencia, CA). After RNA quantification (Nanodrop 1000, Thermoscientific, Wilmington, DE), the cDNA was transcribed from 100 ng of RNA (Qiagen), and then used for quantitative reverse transcription-PCR (qRT-PCR) (StepOnePlus instrument; Applied Biosystems, Carlsbad, CA). SYBR Green-based primers were used (Qiagen). Table 2A shows the GenBank Accession numbers and functions for 23 genes that were investigated. Cancer-related genes were Type 1 Cell-Surface Receptor for TGF-beta ligand superfamily (*ALK1*), Cytochrome P450, DNA Repair associated (*BRCA1*), Family 51, Subfamily A, Polypeptide 1 (*CYP51A*), Dehydrogenase/Reductase Member 2 (*DHRS2/HEP27*), Epidermal Growth Factor Receptor (*EGFR*), Erb-b2 Receptor Tyrosine Kinase 2 (*ERBB2*), Excision Repair Cross-Complementation Group 1 (*ERCC1*), and Secreted frizzled-related protein 1 (*SFRP1*). We also examined genes involved in cisplatin-induced nephrotoxicity and resistance (26, 27): ATP-Binding Cassette, Sub-Family C (*ABCC*1), Eukemia viral BMI-1 oncogene (*BMI1*), Cyclin-dependent kinase 2 (*CDK2*), Cyclin-dependent kinase inhibitor 1A (*CDKN1A*/*P21*), Lysine acetyltransferase 5 (*KAT5/TIP60*), and Tumor protein p53 (*TP53*).

Apoptosis genes were BCL2-associated X protein (*BAX*), BCL2 Binding Component 3 (*BBC3*), BCL2-Like-13 (*BCL2L13*), Caspase-3 (*CASP3*), and Caspase-9 (*CASP9*). Signaling genes include EGF-Containing Fibulin-Like Extracellular Matrix Protein-1 (*EFEMP1*), Mitogen-activated protein kinase 8 (*MAPK8*), Mitogen-activated protein kinase 10 (*MAPK10*), and Forkhead Box M1 (*FOXM1*). Target cycle thresholds (Ct) values were initially compared to the Ct values of reference genes and subsequently, comparisons between untreated and cisplatin-treated values (ΔΔCt) were evaluated for statistical significance. Fold differences were calculated using the equation 2^(ΔΔ*Ct*)^.

### Statistical Analyses

Statistical analysis of the data was performed by ANOVA (GraphPad Prism, version 5.0). Newman-Keuls multiple-comparison or the two-tailed *t*-tests were used to compare the data within each experiment. *P* ≤ 0.05 was considered statistically significant. Error bars in the graphs represent standard error of the mean (SEM).

## Results

### Sequencing of mtDNA From H and J Cybrids

The entire mtDNA from the J and H cybrids were sequenced using NGS technology. Figure 1A shows the age and gender of person in this study. The private SNPs are those that do not define the J or H haplogroups (non-haplogroup defining). The unique SNPs are not listed in www.MitoMap.org or other programs. Table 1A shows the SNPs in the J haplogroup cybrids. There were 11 private SNPS in the mtDNA regions of the J cybrids: *C*YB 10-01 with m.2305T>C (unique, no rs#, MT-RNR2, unique) and m.10654C>T (no rs#, MT-ND4L, Ala62Val); CYB 10-05 with m.7226G>A (rs369835151, MT-CO1, Syn:Ser441); m.13143 (rs386829174, MT-ND5, Syn:Asn269); and with m.16209T>C (rs386829278, MT-CR, NonCoding); CYB 11-32 with m.6734G>A (rs41413745, MT-CO1, Syn:Met332); CYB 13-43 with m.3847T>C (no rs#, MT-ND1, Syn:Leu181); m7805G>A (no rs#, MT-CO2, Val74Ile) and m.14208A>G (no rs#, MT-ND6, Thr156Ala); m.16263T>A (rs386829294, CR;HV1, NonCoding); CYB 13-62 with m.6899GA (no rs#, MT-CO1, Syn:Met332); and CYB 13-107 with m.8200T>C (no rs#, MT-CO2, Syn:Ser205) (Table 1A). The non-coding Control Region (CR) of the J cybrids possessed 17 SNPs and 10 of those defined the J haplogroup. The NGS methodology allowed identification of heteroplasmic SNPs in the J cybrids. The 13 heteroplasmy SNPs showed a range from 3.1 to 17.29% occurrence in five of the seven cybrids. CYB 10-05 and CYB 13-74 lacked heteroplasmy in the mtDNA.

**Table 1A:** SNPs found by NGS in J Haplogroup Cybrids.

**Loci: MT-**	**SNP**	**AA Change**	**Locus**	**rs#**	**CY 10-01**	**CY 10-05**	**CY 11-32**	**CY 12-43**	**CY 13-62**	**CY 13-74**	**CY 13-107**
CR:HV2/OHR	152 T>C		NonCoding	rs117135796	J1d						
CR:HV2/OHR	185 G>A		NonCoding	na		J1c	J1c				
CR:HV2/OHR	200 A>G		NonCoding	rs372099630							8.28% Htroplsmy
CR:HV2/OHR	228 G>A		NonCoding	rs41323649		J1c	J1c			J1c	
CR:HV2/OHR	242 C>T		NonCoding	na					J1b1a		
CR:HV2/OHR	271 C>T		NonCoding	na				J1b1b			
CR:HV2/OHR	295 C>T		NonCoding	rs41528348	J	J	J	J	J	J	J
CR:HV3	462 C>T		NonCoding	rs41402146	J1	J1	J1	J1	J1	J1	J1
CR:HV3	482 T>C		NonCoding	rs386419941		J1c1				J1c1	
CR:HV3	489 T>C		NonCoding	rs28625645	J	J	J	J	J	J	J
tRNA Phe	596 T>C		tRNA	na							5.4% Htroplsmy
RNR1	1007 G>A		rRNA	rs111033213	J1d1a						
RNR2	2158 T>C		rRNA	rs41349444					J1b1a		
RNR2	2305 T>C	Unique		na	PVT-b						
RNR2:Hum	2655 G>A	Unique		na				7.95% Htroplsmy			
RNR2	3010 G>A		rRNA	rs3928306	J1	J1	J1	J1	J1	J1	J1
RNR2	3197 T>C		rRNA	rs2854131							6.79% Htroplsmy
TER/TL1	3242 G>A		tRNA	rs193303018							17.29% Htroplsmy
ND1	3394 T>C	Tyr30His		rs41460449		J1c1				J1c1	
ND1	3847 T>C	Syn:Leu181		na				PVT-a			
ND1	4216 T>C	Tyr304His		rs1599988	JT	JT	JT	JT	JT	JT	JT
ND2	5460 G>A	Ala331Thr		rs3021088				J1b1	J1b1		
tRNA Cys	5821 G>A		tRNA	rs200587831	3.44% Htroplsmy						
CO1	6261 G>A	Ala120Thr		rs201262114		PVT-a					
CO1	6554 C>T	Syn:Thr217		na			J1c7				
CO1	6734 G>A	Syn:Met277		rs41413745			PVT-b				
CO1	6899 G>A	Syn:Met332		na					PVT-b		
CO1	7146 A>G	Thr415Ala		rs372136420	3.25% Htroplsmy						
CO1	7226 G>A	Syn:Ser441		rs369835151		PVT-b					
CO1	7256 C>T	Syn:Asn451		rs41542322	4.2% Htroplsmy			3.1% Htroplsmy			
CO2	7747 C>T	Syn:Asn54		rs28608702		J1b1b1c					
CO2	7789 G>A	Syn:Leu68		rs386829014	J1d						
CO2	7805 G>A	Val74Ile		na				PVT-b			
CO2	7963 A>G	Syn:Leu126		na	J1d						
CO2	8192 A>G	Asn203Tyr		na							3.24% Htroplsmy
CO2	8200 T>C	Syn:Ser205		na							PVT-b
CO2	8269 G>A	Syn:Ter228		na				J1b	J1b		J1b
ATP8/ATP6	8557 G>A	Syn:Leu64/Ala11Thr		rs386829040					J1b1a		
CO3	9635 A>C	Syn:Ser143		na						J1c1a	
ND3	10398 A>C	Thr114Ala		rs2853826	J	J	J	J	J	J	J
tRNA Arg	10410 T>A		tRNA	rs200478835				J1b1b1			
ND4L	10654 C>T	Ala62Val		na	PVT-b						
ND4	11251 A>G	Syn:Leu164		rs3915952	JT	JT	JT	JT	JT	JT	JT
ND4	11623 C>T	Syn:Tyr288		na						J1c1a	
ND4	11827 T>C	Syn:Ala356		rs368026942					10.16% Htroplsmy		
ND4	12007 G>A	Syn:Trp416		rs2853497					J1b1a		
ND4	12127 G>A	Syn:Gly456		na			J1c7				
ND5	12612 A>G	Syn:Val92		rs28359172	J	J	J	J	J	J	J
ND5	13143 T>C	Syn:Asn269		rs386829174		PVT-b					
ND5	13708 G>A	Ala458Thr		rs28359178	J	J	J	J	J	J	J
ND5	13879 T>C	Ser515Pro		rs200380057				J1b1	J1b1		
ND5	13899 T>C	Syn:Tyr521		rs370031192						J1c1a	
ND6	14028 A>G	Syn:Lys564		na				PVT-b			
CYB	14798 T>C	Phe18Leu		rs28357681		J1c	J1c			J1c	
CYB	15452 C>A	Leu236Ile		rs527236209	JT	JT	JT	JT	JT	JT	JT
CYB	15769 A>G	Syn:Gln341		na			4.9% Htroplsmy				
CR:HV1	16069 C>T		NonCoding	rs147903261	J	J	J	J	J	J	J
CR:TAS2	16092 T>C		NonCoding	na			J1c7				
CR:TAS2	16126 T>C		NonCoding	rs147029798	JT	JT	JT	JT	JT	JT	JT
CR:7SDNA	16145 G>A		NonCoding	rs41419246				J1b	J1b		J1b
CR:HV1	16193 C>T		NonCoding	na	J1d						
CR:HV1	16209 T>C		NonCoding	rs386829278		PVT-b					
CR:HV1	16222 C>T		NonCoding	rs386829282				J1b			
CR:HV1	16261 C>T		NonCoding	rs138126107				J1b	J1b		J1b
CR:HV1	16263 T>A		NonCoding	rs386829294			J1c7	PVT-a			
CR:HV1	16292 C>T		NonCoding	rs144417390					PVT-a		
CR:HV1	16300 A>G		NonCoding	na	J1d1						
CR:HV1	16309 A>G		NonCoding	rs373517769	J1d1a						

*PVT-a, found in other J or H haplogroups*.

*PVT-b, not found in other J or H haplogroups*.

*na:, not available*.

*All SNP's had a Quality (A Phred-scaled quality score assigned by the variant caller) Score of 100 and PASSed all the Filters*.

Table 1B shows the SNPs in the H haplogroup cybrids. There were 12 private SNPS in the mtDNA regions of the H cybrids: CYB 10-03 with m.1198A>G (no rs#, MT-RNR1), m.1477T>C, (no rs#, MT-RNR1) m.4483C>G (unique, no rs#, MT-ND2), m.9305G>A, (no rs%, MT-CO3, Syn:Met33), m.9771T>C, (unique, no rs#, MT-CO3, Ser189Pro); m.16093T>C (rs2853511, CR:TAS2, NonCoding); CYB 11-23, with m.1750G>A, (rs28491689, MT-RNR2); m.3010G>A (rs3928306, MT-RNR2); m.11587C>T, (no rs#, MT-ND4, Syn:Cys276); CYB 11-35 with m.3010G>A, (rs3928306, MT-RNR2); and CYB 13-49 with m.13911A>G, (no rs#, MT-ND5, Syn:Met525); and m.14025T>C (no rs#, MT-ND5, Syn:Pro563). There were 8 SNPs in the non-coding Control Region with seven of those defining the H haplogroup. Four of the cybrids (CYB 10-07, CYB 11-10, CYB 11-23, and CYB 13-49) possessed heteroplasmy that ranged from 4.39 to 14.22%. The SNP variants associated with human diseases are listed in Table 1C. The m.3010G>A variant (found in all seven of the J haplogroup cybrids and three of the H haplogroup cybrids) is associated with cyclic vomiting syndrome and migraines. The other SNP variants listed are found in either the J cybrids or the H cybrids but not both.

**Table 1B:** SNPs found by NGS in H Haplogroup Cybrids.

**Loci: MT-**	**SNP**	**AA Change**	**Locus**	**rs#**	**CY 10-03**	**CY 10-04**	**CY 10-07**	**CY 11-10**	**CY 11-23**	**CY 11-35**	**CY 13-49**
CR:HV2	73 G>A		NonCoding	rs3087742	H	H	H	H	H	H	H
CR:HV2/ OHR	195 T>C		NonCoding	rs2857291				H4a1a+195			
CR:HV3	456 C>T		NonCoding	rs41356551		H5'36					
RNR1	750 A>G		rRNA	rs2853518	H2a		H2a				
RNR1	961 T>G		rRNA	rs3888511					H11a		
RNR1	1198 A>G		rRNA	na	PVT-b						
RNR1	1438 A>G		rRNA	rs2001030	H		H				
RNR1	1477 T>C		rRNA	na	PVT-b						
tRNA Val	1630 A>C		tRNA	na		H5					
RNR2	1750 G>A		rRNA	rs28491689					PVT-b		
RNR2	2159 T>C		rRNA	na	H63						
RNR2	2706 A>G		rRNA	rs2854128	H	H	H	H	H	H	H
RNR2	3010 G>A		rRNA	rs3928306					PVT-b	PVT-a	PVT-a
ND1	3926 T>C	Leu207Pro		na							14.22% Htroplsmy
ND1	3992 C>T	Thr229Met		rs41402945				H4			
ND1	4024 A>G	Thr240Ala		rs41504646				H4a			
tRNA Gln	4336 T>C		tRNA	rs193303033		H5a					
ND2	4483 C>G	Ala5Gly		na	PVT-b						
ND2	4733 T>C	Syn:Asn88		na							PVT-a
ND2	4769 A>G	Syn:Met100		rs3021086	H2						
ND2	4896 T>C	Tyr143His		na				4.39% Htroplsmy			
ND2	5004 T>C	Syn:Leu179		rs41419549				H4a			
ND2	5051 A>G	Syn:Leu194		na		H5a1i					
ND2	5301 A>G	Ile278Val		rs199794187							
NC3	5585 G>A		NonCoding	rs386828973					H11a2a2		
CO1	6303 G>A	Gly134Stop		na							5.41% Htroplsmy
CO1	6505 T>C	Val201Ala		rs28371932							12.21% Htroplsmy
CO1	6951 G>A	Val350Met		na				PVT-a			
CO1	7028 T>C	Syn:Ala375		rs2015062	H	H	H	H	H	H	H
CO1	7337 G>A	Syn:Ser478		rs386829005			H66				
CO2	8269 G>A	Syn:Term228		rs8896				H4a1a			
tRNA Lys	8343 A>G		tRNA	na			PVT-a				
ATP8	8448 T>C	Met28Thr		na					H11		
ATP8	8512 A>G	Syn:Lys49		na							
ATP6	9123 G>A	Syn:Leu199		rs28358270				H4			
CO3	9305 G>A	Syn:Met33		na	PVT-b						
CO3	9771 T>C	Ser189Pro		na	PVT-b						
CO3	9777 G>A	Gly191Ser		na			13.99% Htroplsmy				
ND4L	10750 A>G	Asn94Ser		rs372297272				5.88% Htroplsmy			
ND4	11587 C>T	Syn:Cys276		na					PVT-b		
ND4	11719 G>A	Syn:Gly320		rs2853495	H	H	H	H	H	H	H
ND4	12130 T>C	Syn:Phe457		na					9.53% Htroplsmy		
ND5	13759 G>A	Ala475Thr		rs386420019					H11		
ND5	13889 G>A	Cys518Tyr		na				H4a1a3			
ND5	13911 A>G	Syn:Met525		na							PVT-b
ND5	14025 T>C	Syn:Pro563		na							PVT-a
ND6	14365 C>T	Syn:Met73		rs2853815				H4a1			
ND6	14582 A>G	Val31Ala		rs41354845				8.61% Htroplsmy			
ND6	14587 A>G	Asp147Ala		na					H11a2		
CYB	14861 G>A	Ala39Thr		rs2853505				4.96% Htroplsmy			
CYB	15670 T>C	Syn:His308		rs527236211					H11a2a2		
CYB	15833 C>T	Syn:Leu363		rs41504845		H5a1					
CR:TAS2	16092 T>C		NonCoding	na					H11a2		
CR:TAS2	16093 T>C		NonCoding	rs2853511	PVT-a						
CR:7SDNA	16140 T>C		NonCoding	rs3134562					H11a2a		
CR:7SDNA	16153 G>A		NonCoding	rs2853512		H5a1i					
CR:7SDNA	16265 A>G		NonCoding	rs386829295					H11a2a2		
CR:HV1	16293 A>G		NonCoding	rs386828867					H11a		
CR:HV1	16304 T>C		NonCoding	rs386829305		H5					
CR:HV1	16311 T>C		NonCoding	rs34799580					H11		

*PVT-a, found in other J or H haplogroups*.

*PVT-b, not found in other J or H haplogroups*.

*na, not available*.

*All SNP's had a Quality (A Phred-scaled quality score assigned by the variant caller) Score of 100 and PASSed all the Filters*.

**Table 1C:** Diseases associated with SNPs^*^ Identified in the J and H Cybrids.

**Loci: MT-**	**SNP**	**AA Change**	**Locus**	**Cybrids with SNP**	**Disease**
RNR1	961 T>G		rRNA	H 1/7	Possible DEAF Associated
RNR2	3010 G>A		rRNA	J 7/7 H 3/7	Cyclic Vomiting Syndrome with Migraine
TER/TL1	3242 G>A		tRNA	J 1/7	Mitochondrial Myopathy/Maternally inherited Hypertrophic Cardiomyopathy/ Renal Tubular Dysfunction; Myelodysplastic syndrome
ND1	3394 T>C	Tyr30His		J 2/7	Leber Hereditary Optic Neuropathy/Carnitine PalmitoylTranserase Deficiency/High Altitude Adaptation
ND1	4216 T>C	Tyr304His		J 7/7	Leber Hereditary Optic Neuropathy/Insulin Resistance/Possible Adaptive High Altitude Variance
tRNA Q	4336 T>C		tRNA	H 1/7	Alzheimer's Disease/Parkinson's Disease/ Hearing Loss & Migraine/Autism Spectrum/Intellectual Disability
ND2	5460 G>A	Ala331Thr		J 2/7	Alzheimer's Disease/Parkinson's Disease
tRNA C	5821 G>A		tRNA	J ‘1/7	Deaf Helper Mutation/Maternally inherited DEAFness/Aminoglycoside-induced DEAFness
COI	6261 G>A	Ala120Thr		J 1/7	Prostate Cancer/Leber Hereditary Optic Neuropathy
tRNA K	8343 A>G		tRNA	H 1/7	Possible Parkinson's Disease Risk Factor
ND3	10398 A>C	Thr114Ala		J 7/7	Parkinson's Disease Protective Factor/Longevity/Altered Cell pH/Metabolic Syndrome/Breast Cancer Risk/Attention Deficit Hyperactivity Disorder/Cognitive Decline/SpinoCerebellar Ataxia Type 2 Age Onset
ND5	13708 G>A	Ala458Thr		J 7/7	Leber Hereditary Optic Neuropathy/Increased Multiple Sclerosis Risk/Higher Frequency in Parkinson's Disease/Alzheimer's Disease
ND6	14582 A>G	Val31Ala		H 1/7	Leber Hereditary Optic Neuropathy Synergistic 14258A + 14582G
CR:TAS2	16093 T>C		NonCoding	H 1/7	Cyclic Vomiting Syndrome
CR:HV1	16300 A>G		NonCoding	J 1/7	Bipolar Disorder Associated

* *Information obtained from www.MitoMap.org and www.HmtVar.uniba.it*.

### IC50 Analyses Results

**Figure 2:**
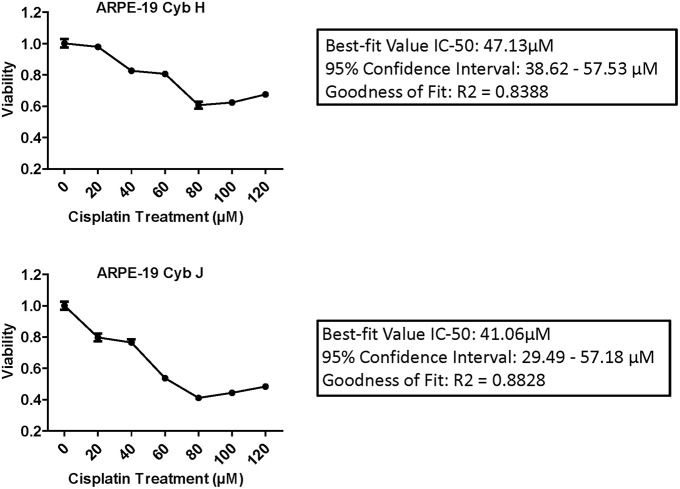
The IC-50 values for the J cybrids were lower (41.06 μM) compared to the H cybrids (47.13 μM). H and J cybrids were plated in 96-well plates, treated with 0, 20, 40, 60, 80, 100, or 120 μM of cisplatin and the cell viabilities measured with MTT reagent. The Goodness of Fit values for the H cybrids were *R*^2^ = 0.8388 and 95% confidence interval 38.62–57.53 μM. The J cybrid Goodness of Fit values were *R*^2^ = 0.8828 and 95% confidence interval 29.49–57.18 μM. Each treatment was analyzed with eight replicates.

IC-50 analyses were performed to determine the concentration of cisplatin required to inhibit the cell viability by 50% (Figure 2). The Goodness of Fit values were *R*^2^ = 0.8388 and *R*^2^ = 0.8828 for the H and J cybrids, respectively. The IC-50 values for cybrid-H were 47.13 μM (95% confidence interval 38.62–57.53 μM) and for cybrid-J were 41.06 μM (95% confidence interval 29.49 and 57.18 μM).

### Viability of H and J Cybrids After Cisplatin Treatment

**Figure 3:**
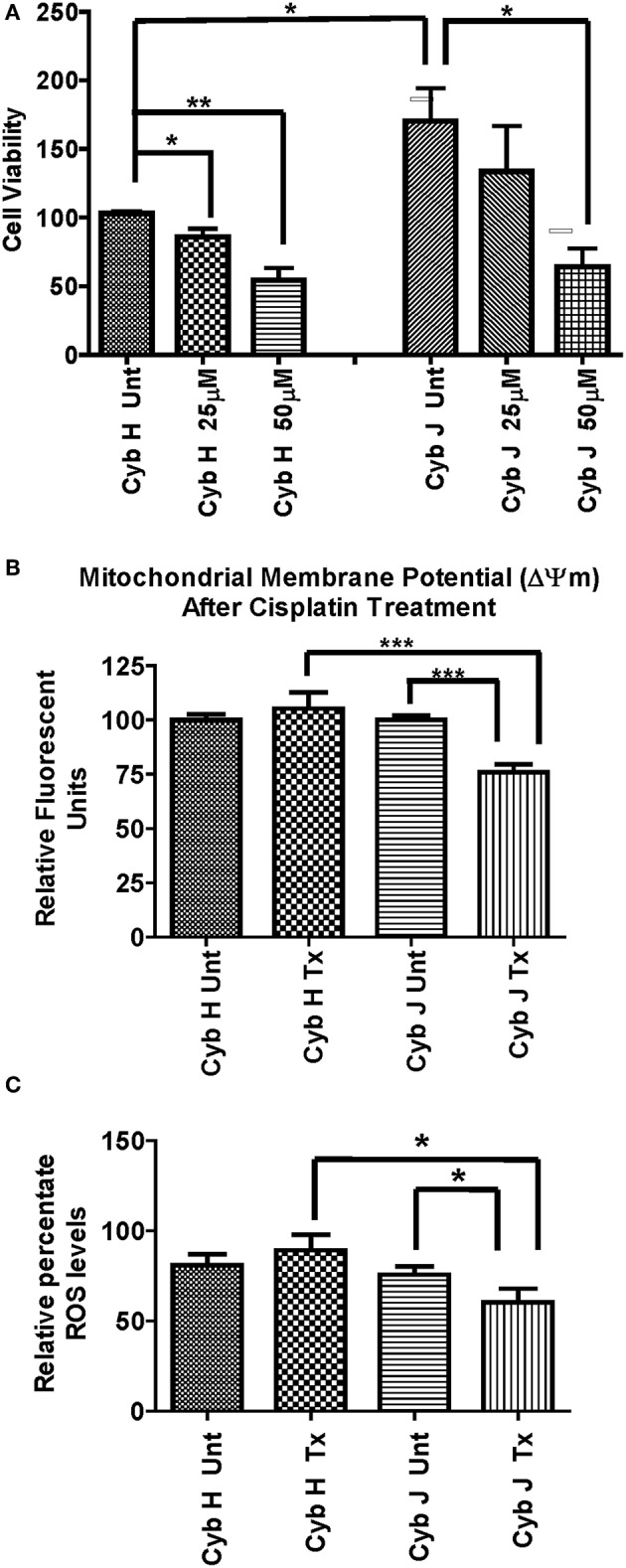
**(A)** The J cybrids were more sensitive to cisplatin treatment than the H cybrid cultures. Cell viabilities of the untreated and cisplatin-treated cybrids were measured using the trypan blue dye exclusion assay and normalized to the untreated-H cybrid sample (Cyb H Unt, 100.0 ± 16.3). The viability in untreated-J cybrids increased at 2.26-fold higher rate than the untreated-H cybrids (*P* = 0.001). The viability declined in the 25 μM cisplatin-treated-H cybrids (13%, *P* = 0.58) and 50 μM cisplatin-treated-H cybrids (38%, *P* = 0.05) compared to the untreated-H cybrids. The viability decreased in the 25 μM cisplatin-treated-J cybrids (35%, *P* = 0.05) and 50 μM cisplatin-treated-J cybrids (65%, *P* = 0.002) compared to the untreated-J cybrids. Each experiment was repeated twice and analyzed in triplicate. **(B)** J cybrids have a greater loss of mitochondrial membrane potential (ΔΨm) after cisplatin treatment than H cybrids. The ΔΨm values in cisplatin-treated-J cybrids were significantly decreased compared to the untreated-J cybrids (*P* = 0.0001) and also the cisplatin-treated H cybrids (*P* = 0.0005). In contrast, the cisplatin-treated and untreated-H cybrids showed similar levels to each other (*P* = 0.51). ΔΨm, mitochondria membrane potential; RFU, relative fluorescent units. ^*^P < 0.05; ^**^P < 0.01; ^***^P < 0.001. **(C)** Cisplatin-treated-H cybrids produced higher levels of ROS compared to the cisplatin-treated-J cybrids. The ROS levels, as measured in relative percentile, were compared between the H and J cybrids without and with cisplatin treatments after 48 h. The cisplatin-treated-J cybrids had significantly lower ROS levels compared to the untreated-J cybrids (*P* = 0.03) and also compared to the cisplatin-treated-H cybrids (*P* = 0.006). The ROS levels for the untreated H and cisplatin-treated H were similar to each other (*P* = 0.37). RFU = relative fluorescent units. ^*^P < 0.05.

The H and J cybrids were treated for 48 h with either 0, 25, or 50 μM cisplatin and cell viabilities measured using a Trypan Blue dye exclusion assay. The cell viability for the untreated J cybrid (Cyb J Unt) was normalized to the untreated H cybrids (Cyb H Unt, 100.0% ± 16.3). Without treatment, the J cybrids grew at a faster rate than H cybrids (226.5% ± 30.7 vs. 100.0% ± 16.3, *P* = 0.001, Figure 3A). Cell viability of H cybrids decreased 13% (*P* = 0.58) after 25 μM cisplatin treatment and to 38% (*P* = 0.05) after 50 μM cisplatin treatment compared to untreated-H cybrids (100.0% ± 16.3). Compared to untreated-J cybrids, viability decreased 35% (*P* = 0.04) and 65% (*P* = 0.002) for the 25 and 50 μM cisplatin-treated-J cybrids, respectively. Thus, cell viabilities of J cybrids were more sensitive to cisplatin treatment than those of H cybrids.

### Mitochondrial Membrane Potential (ΔΨm) After Cisplatin Treatment

The effects of cisplatin on the ΔΨm of H and J cybrids were analyzed after 48 h incubation (Figure 3B). The cisplatin-treated-H cybrids showed similar ΔΨm compared to the untreated-H cybrids (105.3% ± 7.45 vs. 100.0% ± 2.6, *P* = 0.51, respectively). The cisplatin-treated-J cybrids showed a significant reduction of ΔΨm compared to the untreated-J cybrids (75.9% ± 3.6 vs. 100.0% ± 2.0, *P* = 0.0001, respectively). In addition, cisplatin-treated-J cybrids showed a 29.4% decrease in Relative Fluorescent Units (RFU) compared to cisplatin-treated-H cybrids (75.9% ± 3.6 vs. 105.3% ± 7.5, *P* = 0.0005). These findings indicate that J cybrids have a greater loss of ΔΨm after cisplatin treatment than H cybrids.

### Reactive Oxygen Species (ROS) Production After Cisplatin Treatment

The ROS levels, measured in RFU, were compared between the H and J cybrids with and without cisplatin treatments after 48 h incubation (Figure 3C). The cisplatin-treated-J cybrids showed significantly lower ROS compared to the untreated-J cybrids (56.79% ± 7.731 vs. 78.33% ± 4.24, *P* = 0.03, respectively) and also compared to cisplatin-treated-H cybrids (98.26% ± 8.66, *P* = 0.03). There was no difference between the cisplatin-treated-H cybrids and untreated-H cybrids after 48 h incubation (*P* = 0.37). Since ROS production levels had been normalized to cell viability for each condition, our findings showed that after cisplatin treatment, the J cybrid cultures showed significantly less ROS production compared to H treated cybrid cultures.

### Gene Expression Levels in H and J Cybrids Treated With Cisplatin

#### Cancer-Related Pathway Genes

The *CYP51A* gene expression levels were similar in untreated-H and untreated-J cybrids (1.14 ± 0.14-fold, *P* = 0.63, Table 2B). However, after cisplatin treatment, the J-treated cybrids showed increased transcription levels compared to untreated-J cybrids (1.94 ± 0.12-fold, *P* = 0.0057) while the cisplatin-treated-H cybrids were similar to untreated-H cybrids (*P* = 0.12).

**Table 2A:** Description of genes analyzed in cisplatin treated H and J cybrids.

**Symbol**	**Gene name**	**Genbank accession numbers**	**Functions**
			**CANCER-RELATED**
*ABCC1*	ATP-Binding Cassette, SubFamily C	NM_004996	Known as MRP1 (multidrug resistance protein 1). Member of the ATP binding cassette family that transports molecules across membranes. Mutations in ABCC1 N-glycosylation connected with Cisplatin resistance.
*ALK1*	Type 1 Cell-Surface Receptor for TGF-beta ligand superfamily	NM_000020	Type Cell-Surface receptor for TGF-beta superfamily of ligands. Shares similar domain structures in serine-threonine kinase subdomains with other activing receptor-like kinase proteins.
*BRCA1*	DNA Repair associated	NM_007294	Nuclear Phosphoprotein that acts as a tumor suppressor by maintaining genomic stability. Involved in transcription, DNA repair of double-stranded breaks, and recombination.
*BMI1*	Eukemia viral BMI-1 oncogene	NM_005180	Stem cell marker. Has key role in regulating the proliferative activity of both normal and leukemic stem cells.
*CDK2*	Cyclin dependent kinase 2	NM_001798	Associated with apoptosis. Essential for completion of prophase I during meiotic cell division in male and female germ cells.
*CDKN1A/P21*	Cyclin-dependent kinase inhibitor 1A/ p21	NM_000389 NM_001220777 NM_001220778 NM_078467 NM_001291549	Plays a critical role in the cellular response to DNA damage and cisplatin toxicity. Mediates cell cycle arrest. Is cyto-protective.
*CYP51A*	Cytochrome P450, Family 51, Subfamily A, Polypeptide 1	NM_000786 NM_001146152	Member of the cytochrome P450 enzyme family of monooxygenases.
*DHRS2/* *HEP27*	Dehydrogenase/ Reductase (SDR Family) Member 2	NM_182908 NM_005794	NADPH-dependent dicarbonyl reductase activity. Mitochondrial matrix protein. Inhibits MDM2 and stabilizes p53.
*EGFR*	Epidermal Growth Factor Receptor	NM_005228	Triggers cell proliferation when bound to epidermal growth factor
*ERBB2*	Erb-b2 Receptor Tyrosine Kinase 2	NM_004448	Member of epidermal growth factor receptor family of receptor tyrosine kinases. Stabilizes binding of epidermal growth factor to receptor
*ERCC1*	Excision Repair Cross-Complementation Group 1	NM_001166049 NM_001983 NM_202001	Nucleotide Excision Repair formed by electrophilic compounds such as Cisplatin. Forms a heterodimer with XPF endonuclease. Involved in recombination DNA repair, inter-stand crosslink, and lesion repair.
*KAT5/* *TIP60*	K (Lysine) acetyltransferase 5	NM_001206833 NM_006388 NM_182709 NM_182710	Drug resistance; has role in acetylation of histones; modulates DNA damage response.
*SFRP1*	Secreted frizzled-related protein 1	NM_003012	Soluble modulators of Wnt signaling. Regulates cell growth and differentiation. Silencing leads to deregulated pathway, associated with cancer.
*TP53*	Tumor protein p53	NM_000546 NM_001126112 NM_001126113 NM_001126114 NM_001126115 NM_001126116 NM_001126117 NM_001126118 NM_001276695 NM_001276696 NM_001276697 NM_001276698 NM_001276699 NM_001276760 NM_001276761	Regulates genes that induce cell cycle arrest, apoptosis, senescence, DNA repair, or changes in metabolism. Induces apoptosis.
			**APOPTOSIS**
*BAX*	BCL2-Associated X Protein	NM_001291429 NM_001291428 NM_001291430 NM_138761 NM_004324 NM_138764 NM_001291431 NM_138763	Associates to form a heterodimer with BCL2. Functions in apoptotic behavior by opening the mitochondrial voltage dependent anion channel, leading to loss of membrane potential, and opening of cytochrome C.
*BBC3*	BCL2 Binding Component 3	NM_001127240 NM_001127241 NM_014417	Member of the BCL-2 family. Induces mitochondrial membrane permeablization and apoptosis.
*BCL2L13*	BCL2-Like-1	NM_015367 NM_001270729 NM_001270731 NM_001270732 NM_001270734 NM_001270735	BCL2 isoform. Mitochondrial-localized protein. Overexpression results in apoptosis.
*CASP3*	Caspase-3	NM_004346 NM_032991	Effector caspase; Activated by caspases 8, 9, and 10. Effects caspases 6, 7, 9. Belongs to family of proteases involved in apoptosis; Synthesized as inactive precursors and therefore need activation.
*CASP9*	Caspase-9	NM_001229 NM_032996	Part of the apoptosome protein complex formed during apoptosis. Mitochondrial caspase activation.
			**SIGNALING**
*EFEMP1*	EGF-Containing Fibulin-Like Extracellular Matrix Protein-1	NM_001039349 NM_001039348	Fibulin family of extracellular matrix glycoproteins. Binds to epidermal growth factor receptors causing phosphorylation and signaling. Associated with drug resistance and cancer prognosis.
*FOXM1*	Forkhead Box M1	NM_001243088 NM_001243089 NM_021953 NM_202002 NM_202003	Transcriptional activator involved in cell proliferation. Regulates expression of several cyclins. Role in DNA break repairs. Phosphorylated and inactivated during mitosis.
*MAPK8*	Mitogen-activated protein kinase 8	NM_002750 NM_139046 NM_139047 NM_139049 NM_001278547 NM_001278548	Targets specific transcription factors. Mediates immediate-early gene expression. Involved in UV radiation induced apoptosis.
*MAPK10*	Mitogen-activated protein kinase 10	NM_002753 NM_138980 NM_138982	Regulatory role in signaling pathways during neuronal apoptosis; Inhibited by cyclin dependent kinase 5.
			**HOUSEKEEPERS**
*ALASV1*	5'-amino-levulinate synthase 1	NM_000688	Mitochondrial enzyme catalyzes rate-limiting step in heme biosynthesis. Level of the mature encoded protein regulated by heme.
*GUSB*	Glucuronidase, beta	NM_000181 NM_001284290 NM_001293104 NM_001293105	Belongs to the glycosidase family of enzymes that break down complex carbohydrates.
*HMBS*	Hydroxy-methylbilane synthase	NM_000190 NM_001024382 NM_001258208 NM_001258209	Third enzyme of heme biosynthetic pathway; Catalyzes condensation of four porphobilinogen molecules into hydroxymethylbilane.
*HPRT1*	Hypoxanthine phosphor-ribosyl-transferase 1	NM_000194	Transferase catalyzes conversion of hypoxanthine to inosine monophosphate and guanine to guanosine monophosphate.
*TUBB*	Tubulin beta class I	NM_178014 NM_001293213	Part of protein superfamily of globular proteins. Major component of microtubules.

**Table 2B:** Expression levels in cisplatin treated H and J cybrids.

**Symbol**	**Untreated** **Cybrids** **H^**#**^ vs. J** ***P* value, Fold**	**H Cybrids Untreated^**#**^ vs Treated** ***P* value, Fold**	**J Cybrids** **Untreated^**#**^ vs.** **Treated** ***P* value, Fold**	**Treated** **Cybrids** **H^**#**^ vs. J** ***P* value, Fold**
**CANCER-RELATED GENES**				
*ABCC1*	0.35, 0.77 ± 0.19	0.24, 0.82 ± 0.078	0.21, 0.63 ± 0.095	**0.02**, 0.56 ± 0.084
*CDKN1A/P21*	0.61, 1.41 ± 0.66	**0.002**, 4.89 ± 0.51	0.12, 3.12 ± 0.65	0.13 0.68 ± 0.14
*CYP51A*	0.63, 1.14 ± 0.14	0.12, 2.01 ± 0.47	**0.0057**, 1.94 ± 0.12	0.75, 1.14 ± 0.07
*DHRS2/HEP27*	0.47, 0.79 ± 0.17	0.89, 1.08 ± 0.14	0.75, 1.12 ± 0.42	**0.02**, 0.53 ± 0.06
**APOPTOSIS GENES**				
*BAX*	0.97, 1.01 ± 0.09	0.32, 1.32 ± 0.26	**0.05**, 1.78 ± 0.26	0.28, 1.4 ± 0.21
*CASP3*	0.63, 1.14 ± 0.14	0.12, 2.01 ± 0.47	**0.02**, 3.33 ± 0.62	0.57, 1.4 ±0.26
**SIGNALING GENES**				
*EFEMP1*	0.57, 0.79 ± 0.32	0.14, 0.69 ± 0.16	0.34, 0.43 ± 0.21	**0.04**, 0.31 ± 0.08
*SFRP1*	0.49, 0.97 ± 0.30	0.28, 0.53 ± 0.26	**0.05**, 0.14 ± 0.04	0.19, 0.77 ± 0.21

*Fold values >1 indicate up-regulation of the gene*.

*Fold values <1 indicate down-regulation of the gene*.

# *are assigned a value of 1*.

*P values ≤ 0.05 are statistically significant and are bolded*.

Levels of *CDKN1A/p21* were similar in untreated-J and H cybrids (*P* = 0.61) but the cisplatin-treated-H cybrids showed significant upregulation compared to the untreated-H cybrid (4.89 ± 0.51-fold, *P* = 0.002; Table 2B). The *CDKN1A/p21* levels in cisplatin-treated-J cybrids trended higher, but were not significant (3.12-fold, *P* = 0.12). Gene expression levels of both *ABCC1* and *DHRS2/HEP27* in cisplatin-treated-J cybrids were significantly lower than that of cisplatin-treated-H cybrids (0.56 ± 0.084-fold, *P* = 0.02 and 0.53 ± 0.06-fold, *P* = 0.02, respectively). The cisplatin-treated-J cybrids had decreased *SFRP1* gene expression levels compared to untreated-J cybrids (0.14 ± 0.04-fold, *P* = 0.05). In contrast, the untreated-H and cisplatin-treated-H cybrids were *SFRP1* levels were similar to each other (*P* = 0.28). These findings demonstrate that expression levels of *ABCC1, CYP51A, CDKN1A/p21, DHRS2/HEP27*, and *SFRP1* genes are differentially influenced in cells containing H vs. J mtDNA profiles.

The levels of *ALK, BRCA1, BMI1, CDK2, EGFR, ERBB2, ERCC1, KAT5/TIP60*, and *TP53* were not different from each other in the H and J cybrids under any conditions (with or without cisplatin treatment; data not shown).

#### Apoptosis Pathway Genes

Untreated-H and untreated-J cybrids expressed similar levels of *BAX* (1.01-fold, *P* = 0.97). After cisplatin exposure, *BAX*, and *CASP3* levels increased in cisplatin-treated-J cybrids compared to the untreated-J cybrids (1.78 ± 0.26-fold, *P* = 0.05 and 3.33 ± 0.62-fold, *P* = 0.02, respectively). In contrast, the treated and untreated-H cybrids were not significantly different from each other (*BAX, P* = 0.32 and *CASP3, P* = 0.12). The *BBC3, BCL2L13*, and *CASP9* gene levels were similar in H and J cybrids with or without cisplatin treatment, indicating that cisplatin did not affect these apoptotic genes. These findings indicate that in the J cybrids, cisplatin-induced apoptosis upregulation of *BAX* and *CASP3*.

#### Signaling Pathway Genes

The cisplatin-treated-J cybrids demonstrated lower *EFEMP1* gene expression levels than cisplatin-treated-H cybrids (0.31 ± 0.08-fold, *P* = 0.04). The *MAPK8, MAPK10*, and *FOXM1* expression levels were not different between H and J cybrids, nor were they affected by cisplatin treatment (data not shown).

### Target Sites for Cisplatin Within the MT-Dloop and Comparison of mtDNA GG Stretches

The entire control regions of H (*n* = 6) and J (*n* = 7) cybrids were sequenced (Table 3) and analyzed for the numbers of GG stretches, which are known to be target DNA sequences for cisplatin (28). The MT-Dloop was analyzed because it is the region controlling replication and transcription for mtDNA. In H cybrids (*n* = 6) and J cybrids (*n* = 7) there were three GGG stretches (nt16455-16457; nt16516-16518, and nt34-36), one GGGGG stretch (nt16470-16474), and one GGGGGG stretch (nt66-71) site. One H cybrid had a GGGG stretch (nt322-325) that was lacking in any of other H or J cybrids. Greatest variability was found in regions of GG stretches: nucleotides (nt) 184-185 (5/7 in J cybrids); nt228-229 (4/7 in J cybrids); nt322-323 (5/6 in H cybrids); nt513-514 (1/6 H cybrids); and nt526-527 (1/6 H cybrids). A difference in GG stretch patterns of H vs. J cybrids may potentially lead to variations in numbers of cisplatin-mtDNA adducts. However, we believe additional studies will be needed to clarify mechanisms of interaction between the mtDNA and cisplatin that might affect cellular responses.

**Table 3:** Total numbers of GG stretches in the MT-Dloop (nt16441 to nt601) of H cybrids vs. J cybrids.

**mtDNA Haplogroup**	**Total nucleotides**	**GG**	**GGG**	**GGGG**	**GGGGG**	**GGGGGG**
H	760	nt16503-16504 (6/6) nt16569-01 (6/6) nt08-09 (6/6) nt53-54 (6/6) nt100-101 (6/6) nt106-107 (6/6) nt184-185 (6/6) nt228-229 (6/6) nt322-323 (5/6) nt409-410 (6/6) nt412-413 (6/6) nt513-514 (1/6) nt526-527 (1/6)	nt16455-16457 (6/6) nt16516-16518 (6/6) nt34–36 (6/6)	nt322-325 (1/6)	nt16470-16474 (6/6)	nt66-71 (6/6)
J	760	nt16503-16504 (7/7) nt16569-01 (7/7) nt08-09 (7/7) nt53-54 (7/7) nt100-101 (7/7) nt106-107 (7/7) nt184-185 (5/7) nt228-229 (4/7) nt322-323 (7/7) nt409-410 (7/7) nt412-413 (7/7)	nt16455-16457 (7/7) nt16516-16518 (7/7) nt34–36 (7/7)	0	nt16470-nt16474 (7/7)	nt66-71 (6/6)

*H, n = 6; J, n = 7*.

## Discussion

### Cell Culture Studies

Although cybrids have identical nuclei and culture conditions, the cell lines with J haplogroup mtDNA exhibit different responses to cisplatin than cybrids with H haplogroup mtDNA. The untreated-J cybrids have significantly increased rates of growth compared to untreated-H cybrids (226 vs. 100%, *P* = 0.001), a finding consistent with a previous study (17). After treatment with cisplatin, J cybrids show a dose-dependent decrease in cell viability with a 35% decline at 25 μM cisplatin (*P* = 0.044) and 58% decline at 50 μM cisplatin (*P* = 0.0023) compared to the untreated-J cultures. In contrast, H cybrids had non-significant 12% decrease at 25 μM and 38% decline at 50 μM (*P* = 0.05) compared to the untreated-H cybrids. The large decline in cell viability for J cybrids may be because cisplatin, similar to many anti-cancer drugs, is more effective on rapidly growing cells (1), which is the status of cells containing J mtDNA haplogroup patterns (17, 29). Alternatively, it may be that the differential effects of cisplatin are related to J cybrids having lower oxygen consumption, ATP levels, and mitochondrial membrane potential (17, 30). Interestingly, Ghelli et al. reported that Leber's Hereditary Optic Neuropathy (LHON) cell lines with the J haplogroup showed increased sensitivity to 2,5-hexanedione (2,5-HD), a toxic solvent that causes neurological and retinal pathology after exposure (31).

The ΔΨm decreased significantly in cisplatin-treated-J cybrids but not in cisplatin-treated-H cybrids compared to their untreated controls. The decline in ΔΨm represents early changes that can lead to downstream events such as apoptosis. The release of cytochrome *C* and induction of intracellular apoptosis are mediated through the voltage-dependent anion channel (VDAC) and cisplatin binds to cysteine and methionine sites of the VDAC (32). The mitochondrial size, shape, and degree of fragmentation can affect binding capacity of BAX, causing changes in mitochondrial outer membrane permeability and apoptosis. The lower ΔΨm levels in cisplatin-treated-J cybrids are consistent with qRT-PCR results showing increased apoptotic gene expressions (*BAX* and *CASP3*) compared to untreated-J cybrids, while the levels in H cybrids did not vary after cisplatin treatment. Our findings show that the mtDNA variants within cells can mediate cisplatin-induced pro-apoptosis events that might contribute to the degrees of toxicity and/or resistance in different individuals.

The cisplatin-treated-H cybrids showed higher ROS levels compared to the cisplatin-treated-J cybrids, which is not completely surprising because the H cybrids utilize OXPHOS, a system that can generate endogenous ROS, while J cybrids use predominantly glycolysis (17). In addition, there are reports that cisplatin reduces mitochondrial respiration complexes I-IV activity by 15–55%, resulting in higher ROS generation in porcine proximal tubular cells (33). A similar stimulus of ROS production may occur in H cybrids because of their reliance on the OXPHOS bioenergetics.

The RPE cell line used in this study is non-cancerous (ARPE-19) and cancer cells genomes may respond differently to cisplatin treatment. One side effect of cisplatin therapy is mild to moderate pigmentary retinopathy (abnormalities of the RPE cells) that occurs in some patients but not in others. Our findings demonstrate that *in vitro* the human RPE cells are affected deleteriously by cisplatin treatment but depending upon the mtDNA haplogroup (H vs. J), the responses are differentially expressed. This differential response may contribute to the pigmentary retinopathy found in some patients but lacking in other subjects (7).

### Gene Expression Studies

#### Differentially Expressed Genes

We looked at several genes involved with apoptosis: *BAX, BBC3, BCL2L13, CASP9*, and *CASP3*. RNA levels for *BAX* and *CASP3* were upregulated in cisplatin-treated-J cybrids compared to untreated-J cybrids, but the H treated cybrids remained similar to untreated controls. Cisplatin-induced upregulation of *CASP3*, the downstream effector gene for apoptosis, has also been reported in carcinoma cells (34). Consistent with elevated apoptosis genes, RNA levels for *BAX* were upregulated after cisplatin treatment in J cybrids in comparison to untreated-J cybrids. *BAX* proteins help form porous defects in the mitochondrial outer membrane, leading to release of apoptotic factors (26). In a mouse model, cisplatin has been shown to induce *BAX* in renal tubular cells (35). Mice deficient of *BAX* show less cytochrome *C* release from mitochondria, lower levels of renal tubular apoptosis, and increased resistance to cisplatin (35). Further studies into the mechanisms of *BAX* and *CASP3* upregulation in our cybrids are needed.

*SFRP1* levels were not significantly changed after cisplatin treatment in H cybrids. In contrast, the *SFRP1* levels were lower in cisplatin-treated-J cybrids compared to untreated-J cybrids (*P* = 0.05). This is significant because all nuclei and culture conditions are identical, indicating that cisplatin has different effects on cells depending on whether they contain H or J mtDNA. *SFRP1* is an extracellular inhibitor of the WNT pathway and acts as a tumor suppressor. Lower *SFRP1* levels, as seen in the cisplatin-treated-J cybrids, can be associated with resistance to cisplatin and poorer patient survivability (36).

The *CDKN1/p21* gene levels were higher after cisplatin treatment in H cybrids compared to untreated-H cybrids (*P* = 0.002). In contrast, the *CDKN1/p21* gene levels were not changed in the J cybrids after treatment (*P* = 0.12). Higher levels of *CDKN1/p21* are significant because of its role in cellular responses to DNA damage and cell cycle arrest. Overexpression of *p21* inhibits colony formation of tumor cells (37). In addition, Duensing et al. reported that abnormal expression of *p21* is associated with chromosomal instability as seen in tumor cells (38). Usually, induction of *CDKN1A* mRNA level is dependent on *Tp53* (39) but in our H and J cybrids, the *Tp53* expression levels were not changed in response to cisplatin treatment (data not shown). This suggests that upregulation of *CDKN1A/p21*, as seen in cisplatin-treated-H cybrids, is independent of *Tp53* expression.

There was an interesting disparity in the J and H cybrids after cisplatin treatment. The untreated-H and J cybrids had similar expression levels for *ABCC1, DHRS2/HEP27*, and *EFEMP1* but after cisplatin-treatment, the treated-J cybrids showed lower expression levels compared to the untreated-J cybrids (*P* = 0.02, *P* = 0.02, and *P* = 0.04, respectively), while the cisplatin-treated-H were similar to the H-untreated. After cisplatin treatment, higher expression levels of *DHRS2/HEP27* were found in the cisplatin-treated-H cybrids compared to the cisplatin-treated-J cybrids. An accumulation of DHRS2/HEP27 pre-protein in the mitochondria matrix can lead to mature DHRS2/HEP27 translocating to the nucleus, where it eventually binds and inhibits MDM2, leading to stabilization of the *Tp53* pathway and indirect tumor repressor functions within the cell. Higher levels of *DHRS2/HEP27* gene expression in cisplatin-treated-H cybrids could be associated with MDM2-mediated breakdown of the *Tp53* gene, and ultimately stabilization of cellular homeostasis (40).

*ABCC1* functions as a transporter, mediates export of drugs from the cytoplasm, and also confers resistance to anti-cancer drugs (41, 42). In osteosarcoma cells, *ABCC1* was the most relevant transporter associated with drug resistance, along with the *ABCB1* transporter (27). After cisplatin treatment, up-regulations of *ABCC1* and *EFEMP1* were found in the cisplatin-treated-H cybrids compared to the cisplatin-treated-J cybrids. The higher *ABCC1* levels in H cybrids suggest these cells may be more likely to become resistant to anticancer drugs than cells with J mtDNA. If our *in vitro* findings represent *in vivo* events, then patients with J haplogroup mtDNA might be less likely to become resistant to anticancer drugs while the H haplogroup patients are more likely. *EFEMP1* is a gene associated with the production of EGF-containing fibulin-like extracellular matrix protein 1. Overexpression of this gene has been linked to *in vivo* and *in vitro* chemotherapeutic drug resistance in cases used to treat glioblastoma tumor growth. The higher *EFEMP1* gene expression levels in H cybrids may be a precursor to drug resistant cybrid cells treated with cisplatin (43). Future studies should be conducted to determine if there is correlation between mtDNA haplogroup patterns and cisplatin resistance.

Based upon our findings, it is apparent that after cisplatin treatment, cells containing J haplogroups (Northern European) variants show different biological behavior and gene expression patterns compared to those cells possessing the Southern European H haplogroup mtDNA. At this time, it is not clear how the H vs. J mtDNA profiles influence the nuclear gene expression in cybrids. The effects may be not strictly related to the haplogroup mtDNA as a whole but rather the presence of specific SNPs, which in this case, are defining the J haplogroup. For example, the m10398A>G polymorphism (*MT-ND3*, ALA-THR) defines the J haplogroup (www.MitoMap.org). The m10398A>G has been associated with breast cancer in African-American women (44), although no association was found in another study (45). Another J haplogroup-defining SNP, m13708G>A (*MT-ND5*, ALA-THR), has also been associated with breast cancer (46). Even though these SNPs were located in different mtDNA encoding genes, each of these has an amino acid change from Alanine, a non-polar, neutral amino acid to the polar, neutral Threonine, which could change bonding and function of proteins. In contrast, when mtDNA from H cybrids were sequenced, there were no mtDNA variant/mutations that have been associated with human cancers (47). One can speculate that one or all of these J haplogroup-defining SNPs can contribute to changes in retrograde signaling between the mtDNA and nuclear genomes. While further work is needed to identify mechanisms and pathways, our findings support the hypothesis that an individual's mtDNA background can contribute to the response to cisplatin, which is important for the drug's efficacy, level of side effect and development of resistance.

**Figure 4:**
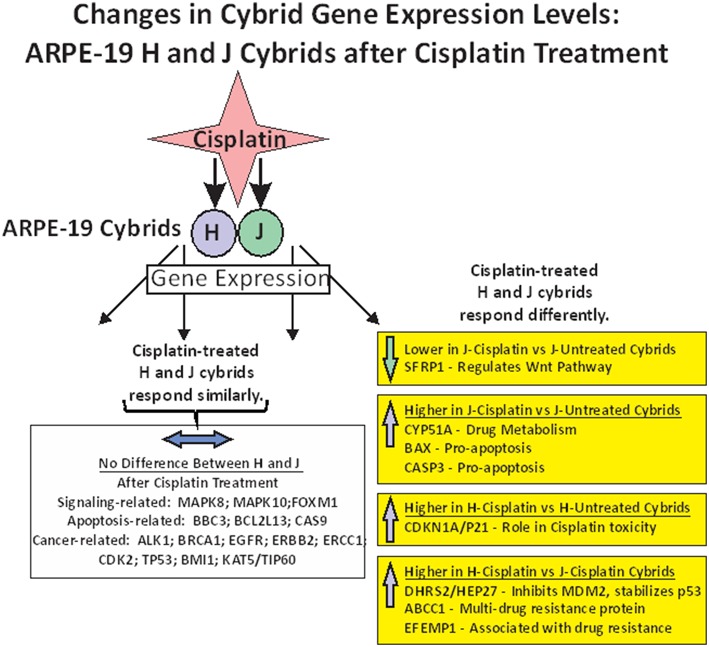
Schematic showing the changes in gene expression levels for H and J cybrids after cisplatin treatment fall into different categories: (1) there are no differences in the expression levels between the untreated and cisplatin-treated cybrids (*MAPK8, MAPK10, FOXM1, BBC3, BCL2L13, CASP9, ALK1, BRCA1, EGFR, ERBB2, ERCC1, CDK2, TP53, BMI1*, and *KAT5/TIP60*). (2) Lower expression levels of *SFRP1* are found in the cisplatin-treated-J cybrid compared to the untreated-J cybrid; (3) Upregulation of *CYP51A, BAX*, and *CASP3* in the cisplatin-treated-J cybrids compared to the untreated-J cybrids; (4) Higher levels of *CDKN1A/P21* are found in the cisplatin-treated H cybrids compared to the untreated H cybrids; (5) The H-cisplatin cybrids showed higher expression levels of *DHRS2/HEP2, ABCC1*, and *EFEMP1* compared to the cisplatin-treated-J cybrids.

In summary of the gene expression studies, the effects of cisplatin on gene expression fell into five categories (Figure 4). The first category was that cisplatin did not change gene expression in either cisplatin-treated-J or cisplatin-treated-H cybrids (*MAPK8, MAPK10, FOXM1, BBC3, BCL2L13, CASP9, ALK1, BRCA1, EGFR, ERBB2, ERCC1, CDK2, TP53, BMI1*, and *KAT5/TIP60*). The second category was that cisplatin-treated-J cybrids were downregulated (*SFRP1*) and the third category showed upregulation (*CYP51A, BAX*, or *CASP3*) compared to the untreated-J cybrids. The fourth category was that cisplatin-treated-H cybrids showed increased transcription of *CDKN1A/p21* gene compared to untreated-H cybrids, while the J cybrid levels were not affected. Finally, that cisplatin-treated-J cybrids showed lower levels of gene expression compared to cisplatin-treated-H cybrids (*DHRS2/HEP27, ABCC1*, and *EFEMP1*).

### Sequencing of the Entire mtDNA

NGS analyses performed on the mtDNA from each of the H and J cybrids showed that the majority of the SNPs identified were haplogroup defining. The private SNPs (non-haplogroup defining), unique SNPs (not listed in www.MitoMap.org), and heteroplasmy SNPs were found in individual cybrids and not throughout all of the H or J cybrids. This suggests that the differential retrograde signaling between the H and J mtDNA haplogroups is due to the accumulation of the haplogroup defining SNPs rather than a single mutation or private SNP. Our findings are consistent with the sequencing results from another cybrid study that used allelic discrimination and Sanger sequencing to identify the mtDNA haplogroups (48). The advancement of NGS for mtDNA analyses allowed deep sequencing in ranges from 1,000 to 100,000 with an average depth of 30,000 so that low level heteroplasmy could be reliably identified. In addition, our method allowed for both strands of mtDNA to be independently sequenced and in both directions, which helps to distinguish between artifact and low level heteroplasmy. The mechanisms of retrograde signaling for the different mtDNA haplogroups are under investigation and likely include as of yet unidentified pathways.

### Comparison of GG Stretches Within the MT-DLoop Region

Our previous report related to the *MT-DLoop* region showed the greatest SNP variations in J vs. H mtDNA were in (a) the nucleotides (np) 263–461 region; (b) Conserved Sequence Block 2 region (np 299-315); (c), H Strand Origin region (np 110-441); (4) Hypervariable Segment 2 region (np 57-372); and (5) the np 310–321 region that had high variability with C insertions (48). However, H and J mtDNA had similar total numbers of CpG and non-CpG methylation sites in the *MT-DLoop*. The mtDNA is a target for epigenetic modifications and altered methylation patterns have been associated with diseases, drug exposure and aging (49–51). However, the degree of mtDNA methylation is still controversial and some have suggested that the methylation levels are very low to absent in mtDNA (52, 53). Cisplatin causes DNA damage by adduct formation at intra-strand d(GpG) crosslink sites. The rate of DNA adduct formation increases in acidic conditions (28, 54) and higher numbers of GG stretches may lead to more binding of cisplatin to the mtDNA. Importantly, our previous bioenergetic studies showed that J cybrids preferentially use glycolysis and have higher levels of extracellular acidification rates (ECAR) than the cybrids with H haplogroup mtDNA (48), leading to the possibility that the H and J mtDNA *MT-DLoop* may possess different levels of GG sites. However, we found that within the *MT-Dloop* regions, the numbers of GG stretches (GG, GGG, GGGG, and GGGGG) were similar in H and J cybrids. Therefore, the environmental microenvironment may be playing a bigger role in cisplatin-related adduct formation rather than the numbers of GG stretches in the mtDNA but further work is needed to clarify this question.

## Ethics Statement

This study was carried out in accordance with the recommendations from the Institutional Review Board (#2003-3131) of the University of California Irvine with written informed consent from all patients. All subjects gave written informed consent in accordance with the Declaration of Helsinki. The protocol was approved by the University of California Irvine.

## Author Contributions

TP designed experiments, interpreted data, wrote manuscript. LN, CL, and KT interpreted data, wrote manuscript. SC, SA, SL, SJ, MM, and DB interpreted data. MC designed experiments, interpreted data. SRA and NU designed and performed the sequencing experiments and interpreted data. MK designed experiments, Interpreted data, wrote manuscript, provided funding resources.

### Conflict of Interest Statement

MK: Discovery Eye Foundation is a 501(c)3 that has supported her mitochondrial research. She serves as a Board Member for DEF. The terms of this arrangement have been reviewed and approved by the University of California, Irvine in accordance with its conflict of interest policies. MK: Consultant for Allegro, Ophthalmics. The remaining authors declare that the research was conducted in the absence of any commercial or financial relationships that could be construed as a potential conflict of interest.
